# Fuzzy Logic and Bio-Inspired Firefly Algorithm Based Routing Scheme in Intrabody Nanonetworks

**DOI:** 10.3390/s19245526

**Published:** 2019-12-13

**Authors:** Hamza Fahim, Wei Li, Shumaila Javaid, Mian Muhammad Sadiq Fareed, Gulnaz Ahmed, Muhammad Kashif Khattak

**Affiliations:** 1 School of Electronic and Information Engineering, Xi’an Jiaotong University, Xi’an 710049, China; hamzafahim@stu.xjtu.edu.cn (H.F.); kashif@stu.xjtu.edu.cn (M.K.K.); 2 School of Computer Science, Shaanxi Normal University, Xi’an 710119, China; shumaila.javed01@gmail.com; 3 School of Management, Xi’an Jiaotong University, Xi’an 710049, China; gulnazahmed@stu.xjtu.edu.cn

**Keywords:** intrabody nanonetworks, fuzzy logic, firefly algorithm, energy-efficient routing protocol, electromagnetic communication in the terahertz band

## Abstract

An intrabody nanonetwork (IBNN) is composed of nanoscale (NS) devices, implanted inside the human body for collecting diverse physiological information for diagnostic and treatment purposes. The unique constraints of these NS devices in terms of energy, storage and computational resources are the primary challenges in the effective designing of routing protocols in IBNNs. Our proposed work explicitly considers these limitations and introduces a novel energy-efficient routing scheme based on a fuzzy logic and bio-inspired firefly algorithm. Our proposed fuzzy logic-based correlation region selection and bio-inspired firefly algorithm based nano biosensors (NBSs) nomination jointly contribute to energy conservation by minimizing transmission of correlated spatial data. Our proposed fuzzy logic-based correlation region selection mechanism aims at selecting those correlated regions for data aggregation that are enriched in terms of energy and detected information. While, for the selection of NBSs, we proposed a new bio-inspired firefly algorithm fitness function. The fitness function considers the transmission history and residual energy of NBSs to avoid exhaustion of NBSs in transmitting invaluable information. We conduct extensive simulations using the Nano-SIM tool to validate the in-depth impact of our proposed scheme in saving energy resources, reducing end-to-end delay and improving packet delivery ratio. The detailed comparison of our proposed scheme with different scenarios and flooding scheme confirms the significance of the optimized selection of correlated regions and NBSs in improving network lifetime and packet delivery ratio while reducing the average end-to-end delay.

## 1. Introduction

The introduction of wireless technology in the healthcare environment is an effort to provide significant convenience and accessibility. Wireless medical technology in medicine, such as body area networks, has already made a significant impact on hospital systems, where placement of sophisticated wireless devices around the body provides ambient assisted living, remote health monitoring and telemedicine services [1,2]. Nanotechnology is further boasting the potential of Intrabody Nanonetworks (IBNNs) for a wide range of medical applications such as healing spinal cord injuries [3], detection of cancer bio-markers [4,5,6], drug delivery [7] and improved diagnosis [8]. IBNNs are composed of miniature size Nano Biosensors (NBSs) that are capable of obtaining fine-grained data with unprecedented accuracy from specific anatomical areas for monitoring and diagnosis purposes. Due to their small size and better electronic properties, these NBSs have the potential to operate inside the human body without interrupting cellular biological function. One of the types of these NBSs is surface plasmon resonance sensors, which have already been deployed for effectively diagnosing various types of cancers and cardiovascular diseases [9,10].

The tremendous potential of IBNNs in revolutionizing healthcare structure is confined by several fundamental limitations including, Nanoscale (NS) communication challenges and inadequate resources of NBSs in terms of energy, storage and computation [9,11]. In the last few years, research communities focused their attention on addressing these primary challenges for realizing the broader scope of IBNNs. In the context of enabling NS communication, electromagnetic communication in the Terahertz Band (THz) has received significant consideration [10,12]. The immense opportunities brought by electromagnetic communication in THz band such as extremely high data communication speed are leading to the development of new electromagnetic-based communication schemes [13,14]. The development of novel schemes for IBNNs also requires an in-depth comprehension of the intense energy constraint of these NBSs for effective outcomes. The extreme energy constraint of these NBSs are elaborated as- a single NBS has a maximum energy of 800pJ and during single transmission of a 120-bit long data packet it consumes about 10% of its available energy [15]. Thereby NBSs are capable of transmitting only 8 data packets with the maximum energy they may harvest. Therefore, energy efficiency is regarded as the key attribute of the communication protocol for prolonging the lifetime of IBNNs. However, fulfilling the demand for increased network lifetime while ensuring continuous data monitoring and transmission for effective diagnosis and treatment is a laborious challenge for severe energy constraint NS devices.

Concerning the requirement of enabling energy-efficient communication in IBNNs past recent years witnessed a few works that explicitly addressed the communication challenges of IBNNs [15,16,17,18]. However, since routing protocols within IBNNs for efficient data collection are at its infancy, therefore, more efforts are required. The presented work is also an effort towards realizing energy-efficient communication for prolonging the lifetime of IBNNs. In this work, we have considered the well-known fact that sensor node consumes their maximum energy during data transmission. Correspondingly, as mentioned before that NBSs consume about 10% of their available energy during one single transmission and accumulating energy consumption of all the NBSs in response to single request messages consequently results in almost 50% consumption of energy resources. The presented work considers this immense decrease in network energy and proposes an alternative approach for the energy-efficient data collection.

In our proposed protocol, we have clustered NBSs into spatially correlated regions and instead of collecting data from all implanted NBSs, an optimum number of NBSs are selected. Avoiding collecting data from all spatially correlated NBSs and selecting the optimized number of NBSs leads to low energy consumption during the data aggregation process. Moreover, ensuring the involvement of enough NBSs for data reporting also helps in achieving a high level of data accuracy. The selection of correlated regions is performed through the Fuzzy Logic-Based Decision System (FLBDS). The underlying motivation behind using fuzzy-based controlled decisions is its high potential for making energy-aware decisions without using complex mathematical modeling. While the selection of NBSs from nominated correlated regions is performed using the bio-inspired firefly mechanism. Firefly algorithm is a meta-heuristic algorithm inspired by the flashing pattern and behavior of fireflies; it determines the optimum solution with low complexity that supports in realizing the goal of low energy consumption during the data collection in IBNNs. The main contributions of our proposed work are underlined as:

1. In this work, we proposed a new FLBDS selection mechanism for correlated region selection. Our proposed FLBDS technique ensures the selection of those correlation regions for data aggregation that has updated information and maximum residual energy. The proposed FLBDS selection resulted in improved information accuracy and stabilized energy consumption in the event area.
2. We proposed a novel bio-inspired firefly algorithm selection mechanism for evading spatial correlation in the reported data. Our proposed bio-inspired firefly algorithm regulates the nomination of NBSs based on their fitness value. The proposed fitness criteria prevent exhaustion of individual NBSs and consume maximum energy resources of NBSs in transmitting crucial information while preventing the transmission of redundant information.
3. Our proposed work is evaluated by carrying out extensive simulations using the Nano-SIM tool that is an emerging tool integrated into NS3 for simulating electromagnetic-based nanonetworks in the THz band [19]. The detailed simulations investigate the performance of our proposed scheme in comparison with different scenarios and the flooding scheme [19]. The different scenarios opted for comparison are briefly explained as; in the first scenario, we involved all the correlated regions (i.e., without employing FLBDS correlated region selection) and only selected optimum number of NBSs using bio-inspired firefly algorithm. Whereas in the second scenario, we selected only information and energy enriched correlated region with the involvement of all the NBSs in response message generation.

We have organized the rest of the paper as follows: Section 2 discusses FLBDS, bio-inspired and existing routing schemes for IBNNs in detail. In Section 3, we thoroughly demonstrate the proposed fuzzy logic and bio-inspired firefly algorithm based routing scheme. Section 4 deals with the performance evaluation, simulation results and provide an extensive comparison with different scenarios and flooding scheme. In Section 5, we finally draw up the conclusion.

## 2. Related Work

Over the past decade, the need for realizing IBNNs has gained considerable attention. The existing work emphasized on the development of novel low-complexity communication protocols for IBNNs. In this section, we briefly discuss the feasibility and scope of FLBDS and bio-inspired firefly algorithms for IBNNs as our proposed protocol combines these concepts for realizing low-complexity routing protocol. Further, we have discussed the advantages and limitations of existing routing protocols in the rest of this section.

### 2.1. Fuzzy Logic-Based Decision Systems (FLBDS)

After FLBDS was proposed in 1965 [20], researchers adapted them in various directions, including expert systems and artificial intelligence. FLBDS are based on simple concepts, consisting of an input stage, a processing stage and an output stage. The input stage maps inputs to the suitable membership functions. The processing stage invokes a set of rules, which generates a result for each input and combines the results of the rules. Finally, the output stage converts the combined result back into a specific control output value [21]. The most common membership functions used in the literature are triangular, trapezoidal and bell curves [22]. FLBDSs attributes such as low complexity, high flexibility and applicability for an uncertain environment raise its support in Wireless Sensor Networks (WSNs). In WSNs, FLBDSs have been used to improve forwarding decisions and quality of service. In the state of the art literature, the use of FLDBS in developing a clustering-based routing algorithm handles more effectively the challenge of routing associated with an uncertain environment. Besides, it also optimizes the selection of routing metric used by enabling the combination and evaluation of diverse parameters in an efficient manner [23,24,25].

In Table 1, we further shed more light on the contribution of the FLBDS on strengthening the performance of WSNs. From Table 1, we comprehend that the low complexity decision process of FLBDS advocates it as a perfect choice for improving the decision process in IBNNs.

### 2.2. Bio-Inspired Based Schemes

The adaptation of nature-inspired solutions in science and engineering has brought great communication benefits. Swarm intelligence based algorithms such as ant colony optimization [36], artificial bee colony [37], firefly algorithm [38] have the capabilities of handling complex problems with simple rules or methods. In WSNs, swarm intelligence based routing protocols utilizes the forging behavior of small insects for solving complex routing problems [39].

In ad-hoc networks, exploitation of self-organizing capabilities of natural species provides scalability, robustness and effectiveness [40]. The last few years also witnessed the scope of bio-inspired approaches in vehicular ad-hoc networks due to their close communication similarity with the natural species. Bio-inspired vehicular ad-hoc networks routing protocols provide better scalability, robustness, low computational complexity and adaptability as compared to traditional vehicular ad-hoc networks routing protocols [41]. Similarly, in the context of the internet of things, bio-inspired routing techniques are nominated as a suitable choice for handling the routing challenge of frequent changing topology and network object diversity [42].

**Figure 1:**
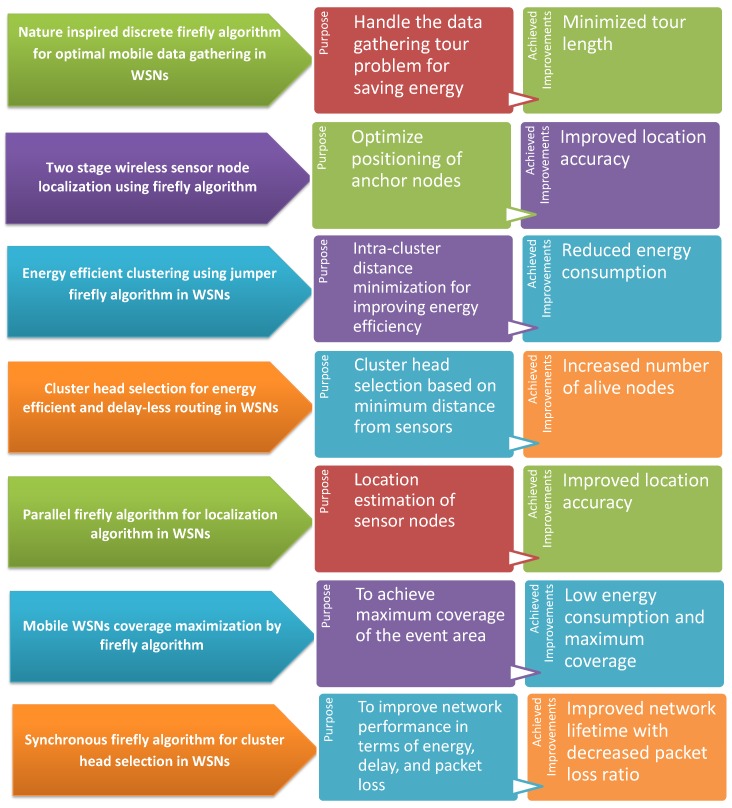
The impact of employing firefly algorithm in improving the performance of WSNs.

Firefly algorithm low complexity among other metaheuristic algorithms has attracted much attention in the last decade [43]. The minimum computation cost of the firefly algorithm in terms of time has led to its adaptation in various applications, including digital image compression [44], antenna design [45], traveling salesman problem [46], security [47,48,49] and multi-modal optimization [50]. In the WSNs, firefly algorithm has also been adapted for handling different problems such as node localization [51,52], energy-efficient data collection [53] and cluster head selection [54,55,56]. From the contributions brought by the firefly algorithm in WSNs, we comprehend that the incorporation of a bio-inspired firefly algorithm in IBNNs ensures a higher probability of achieving low-complexity energy-efficient data routing. In Figure 1, we have further emphasized the impact of the firefly algorithm in improved WSNs performance that is further paving the way for its adaptation in IBNNs.

### 2.3. Network Communication Protocols for IBNNs

Due to the severe constraints of IBNNs regarding energy, transmission range, storage and computational capabilities, the general routing protocols for nanonetworks are not directly applicable in IBNNs. Therefore, in this section, we specifically discuss the existing routing protocol proposed for IBNNs. The selective flooding scheme presented in Reference [19] conserve the limited resources of IBNNs by controlling the direction of forwarded packets for reducing energy consumption and saving bandwidth. In another effort to save energy consumption, a protocol stack for IBNNs is proposed in Reference [15]. The proposed protocol stack introduces two different protocols at the network layer for prolonging the network lifetime. In the greedy scheme, the node with the maximum energy is selected for responding to the request message received from the healthcare monitoring system. The low complexity of the greedy scheme points it as the most suitable approach for conserving energy resources in IBNNs. Another energy conservation scheme presented in Reference [17] introduces nanocluster controllers for performing data aggregation. NBSs transmit data to the closed positioned nanocluster controller that helps in reducing energy consumption. The work presented in Reference [57] thoroughly investigated IBNN and studied the energy harvesting options available for prolonging the life span of NBSs. In the view of the characteristics of the existing energy conservation scheme, we conclude that our proposed scheme differentiates from the state of the art schemes in the following ways:

1. In our proposed work, we select information and energy enriched spatially correlated region for data transmission. While Afsana et al. [17] elects nanocontrollers for aggregating data from their respective layers, which increases the complexity of resource constraint IBNNs. Our proposed FLBDS selection is a low complexity solution for correlated region selection. Moreover, it also reduces the transmission of redundant and invaluable information.
2. In our proposed scheme, we limit the number of transmission of forwarding packets by selecting those NBSs for generating response messages, located at a minimal distance from nanorouter. This selection criterion decreases the possibility of multi-hop communication.
3. Our proposed scheme selects the optimum number of NBSs based on different fitness parameters, the selection of the optimal number of NBSs ensures that data is collected from enough number of NBSs to achieve data accuracy. Whereas the greedy scheme selects only one node for transmitting answer messages based on remaining energy. The selection based on solely differentiates energy while ignoring the distance from the nanorouter results in an increased number of forwarded packets. In addition, data transmission from only one node for energy consumption can also significantly compromise the data accuracy.

## 3. Fuzzy Logic and Bio-Inspired Firefly Algorithm Based Routing Scheme

**Figure 2:**
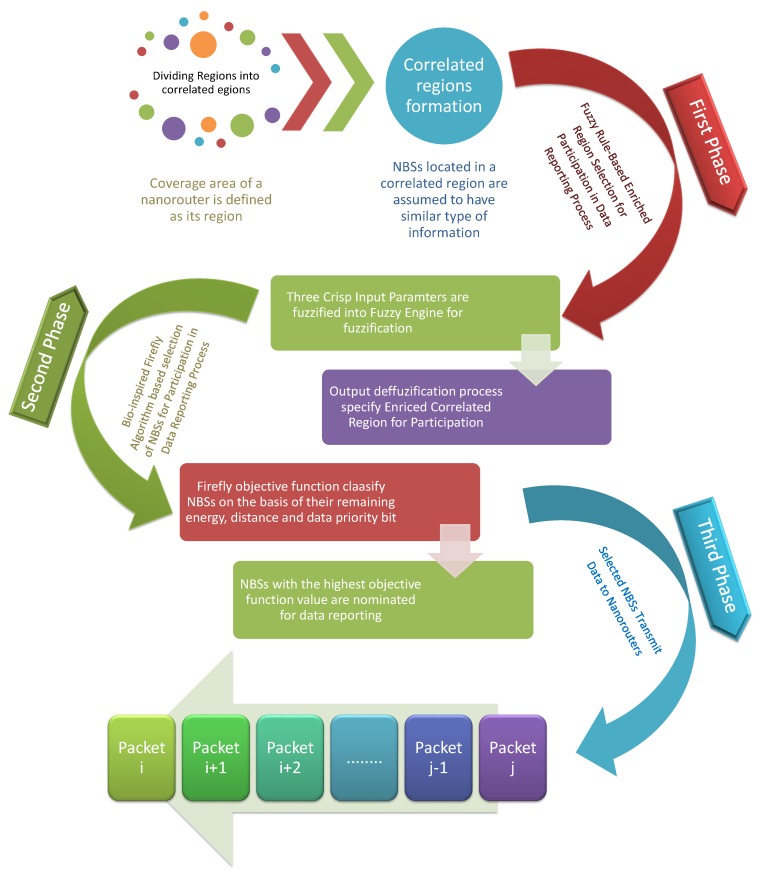
The complete working of the routing scheme proposed in this work.

In this section, we proposed a fuzzy logic and bio-inspired firefly algorithm based routing scheme. The main objective of our proposed scheme is to increase the probability of enriched information transmission while maximizing the lifetime of implanted NBSs. Our proposed scheme realizes the goal of energy-efficient data routing by dividing the data transmission process from NBSs to nanorouter into three different phases, briefly explained in this section. Furthermore, Figure 2 also elaborates on the complete working of our proposed scheme.

- First Phase: In the first phase, nanorouter nominates information and energy enriched regions from the accumulated number of correlated regions—configured during network setup time. The Fuzzy logic-based decision ensures the selection of only those regions that have maximum enrichment values for energy and information. The selection of correlated regions supports in achieving the ultimate goal of important information retrieval with balanced energy consumption. After the selection of participating correlated regions for data reporting, nanorouter broadcast request messages $\left(P_{RqM}\right)$ only for selected correlated regions. Those correlated regions that are excluded from data reporting cannot generate feedback message $\left(P_{FB}\right)$ and response message $\left(P_{RM}\right)$. Avoiding data aggregation from correlated regions that have not valuable information results in evading unnecessary data transmission and saving of energy resources.
- Second Phase: In the second phase, NBSs selection is carried out using the firefly algorithm to support further the objective of reduced redundant data transmission, increased probability of retrieving crucial information and prolonged lifetime of NBSs. Since NBSs located in the correlated regions are assumed to be spatially correlated, which transmit similar types of information. Therefore, the selection of NBSs using the proposed firefly algorithm from selected correlated regions minimize aggregation of the same information. For selecting NBSs, nanorouter determines the fitness of NBSs using the information transmitted by the NBSs in the feedback messages $\left(P_{FB}\right)$. The feedback message $\left(P_{FB}\right)$ contains the information about the priority bit (i.e., the value set to 1 or 0 according to the criticality of the last transmitted message) and residual energy of the NBS. The proposed fitness function ensures the selection of those NBSs for data reporting that have maximum residual energy, minimum distance from the nanorouter and have valuable information. These fitness criteria lead to improved network lifetime of NBSs and increase the probability of receiving important information.
- Third Phase: After selecting NBSs, nanorouter broadcasts announcement message $\left(P_{AM}\right)$ to notify selected NBSs to generate response messages $\left(P_{RM}\right)$. Finally, in the third phase, selected NBSs transmit response messages $\left(P_{RM}\right)$ to the nanorouter.

### 3.1. System Architecture

A possible healthcare monitoring system is composed of an IBNN and an external healthcare system. IBNN consists of two kinds of nanodevices, namely, NBSs and nanorouters implanted inside the human body. NBSs have severe constraints in terms of energy, transmission range, storage and computational resources. While nanorouters have more resources than NBSs and they are responsible for handling more complex operations such as collecting data from NBSs, performing complex computational operations and transmitting collected information to the external healthcare system using nanointerface. Nanointerface is connected to the external healthcare system through the internet for remote data transmission. The communication is carried out until the IBNN is dead.

#### Network Model Assumption and Definitions

We describe our network model as a undirected graph G=(V,E), where *V* denotes set of NBSs and nanorouter (vertices), represented as V∈(nr,nbs) and *E* is the set of edges connecting vertices. In a scenario where an edge is directly connecting nr and $nbs_{i}$ to each other, they are said to be neighbors. The neighbors of router nr are defined as $N\left(nr\right)=\{nr|nr\left(nr,nbs_{i}\right)\in E\}$. The one-hop neighbor set of nr is a symmetric graph $G^{\prime }=\left(V,E\right)$, where given any two pairs of adjacent vertices nr—$nbs_{1}$ and nr-$nbs_{2}$ of *V*, bidirectional edge *E* exist.

**Definition** **1.** *When several NBSs are located in the transmission range of a nanorouter $\left(r_{i}\right)$, a cluster is formed that is expressed as:*

(1) $$Cluster_{members}\left(nr\right)=\left\{\begin{array}{cc} 1 & \mathrm{if}\;\left\{nbs_{1},nbs_{2},\ldots nbs_{n}\right\}\in TR\left(nr\right),\\ 0 & \mathrm{otherwise} \end{array}\right.$$

*where TR(nr) is the transmission radius of the nanorouter nr. Nanorouter nr is responsible for collecting detected information from its cluster members and transmitting it to the external healthcare system.*

**Definition** **2.** *The transmission radius of a nanorouter (nr) is divided into small correlated region, such that the value observed by the NBSs in that spatially correlated region are considered similar (for the application). The correlated region of nanorouter nr are expressed as $\{cr_{1},cr_{2},cr_{3},cr_{n}\}\in nr$. Instead of collecting data from all deployed NBSs, data is aggregated from the optimal number of NBSs, which is sufficient to represent the correlated region $CR_{i}$.*

*The size of the correlation region varies according to the type of application and event characteristics. For those applications that demand a high level of accuracy, the size of the correlation region can be decreased. While for the event characteristics that do not change significantly at a small range, the size of the correlation region can be increased. In this work, the size of the correlated region is defined during the simulation setup time.*

**Definition** **3.** *Participating correlated regions are selected before the data collection process based on the output of the fuzzy logic decision, the detailed selection process is given in the Section 3.2.*

**Definition** **4.** *NBSs selection from participating correlated regions is performed based on the bio-inspired firefly algorithm fitness value. The complete process is demonstrated in the Section 3.3.*

**Definition** **5.** *The lifetime of IBNN is evaluated based on the number of alive NBSs **(m-in-k-of-n nodes)** [58].*

*The total number of NBSs are divided into two groups; directed-neighbors-to-nanorouter and non-directed-neighbors-to-nanorouter denoted as $NBS_{D}$ and $NBS_{ND}$, respectively. According to the **(m-in-k-of-n nodes)** approach, the group of $NBS_{ND}$ are characterized as non-critical NBSs, which means death of k NBSs is allowed from $NBS_{ND}$. While no failure of NBSs from the group of $NBS_{D}$ is tolerable. Accordingly, the network lifetime is elaborated as following:*

*The total number of NBSs available in the network are denoted as $\overset{︷}{NBS}=\{nbs_{1},nbs_{2},\ldots nbs_{n}\}$, the set of all the NBSs that are alive at the certain time t, expressed as U(t).*

(2) $$U(t)=\{u|u\in \overset{︷}{NBS}\wedge u\;alive\;at\;t\},|U(t)|=u(t),$$

*where $u_{i}$ is a NBS that belongs to the set of $\overset{︷}{NBS}$, expressed as $\left(u_{i}\in \overset{︷}{NBS}\right)$, whose energy is not depleted. Now set of NBSs that are part of the $\overset{︷}{NBS_{D}}$ and $\overset{︷}{NBS_{ND}}$ are defined using Equation (3) and (4):*

(3) $$v(t)=\{v|v\in (NBS,\overset{︷}{NBS_{ND}})\wedge v\;is\;non-directed-neighbors-to-nanorouter\;at\;t\},|V(t)|=v(t),$$

*where $\left(v_{i}\in \overset{︷}{NBS_{ND}}\right)$ is a NBS that is a part of $\overset{︷}{NBS_{ND}}$, while v(t) it is also a subset of U(t). Among all the NBSs that are part of $\overset{︷}{NBS_{ND}}$, only k number of NBSs can be dead.*

(4) $$w(t)=\{w|w\in (\overset{︷}{NBS_{D}})\wedge w\;is\;directed-neighbors-to-nanorouter\;at\;t\},|W(t)|=w(t),$$

*where $\left(w_{i}\right)$ is a part of $\overset{︷}{NBS_{D}}$ group denoted as $\left(w_{i}\in \overset{︷}{NBS_{D}}\right)$ and w(t) is also a subset of U(t), death of any NBS from the group $\overset{︷}{NBS_{D}}$ results in the death of the network.*

### 3.2. Fuzzy Logic-Based Correlated Region Selection

In our proposed work, different correlated regions are selected for data reporting; the selection decision of the participating region is performed using FLBDS, as demonstrated in Algorithm 1. Our proposed FLBDS significantly overcomes the complexity overhead of the selection decision of correlated regions in resource constraint IBNNs.

**Figure 3:**
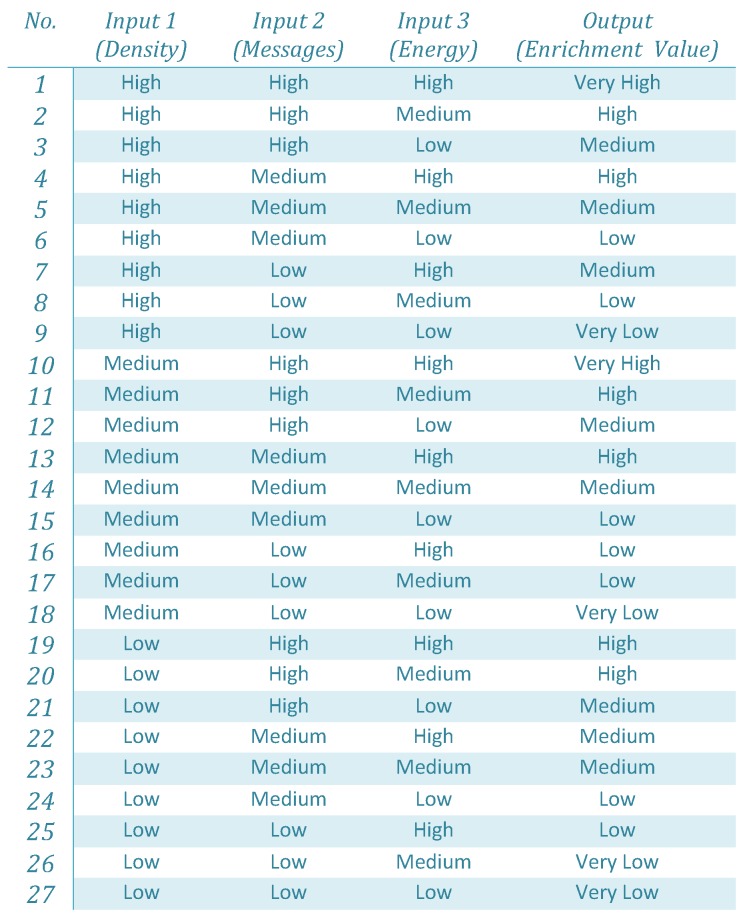
The Mamdani rules used for fuzzy logic-based correlated region selection.

**Algorithm 1:** Selection decision of correlated regions for data reporting using FLBDS  1:
**Input:**
  2:Set of correlated regions $\left\{CR^{r}\right\}=\left\{cr_{1},cr_{2},cr_{3}\ldots cr_{n}\right\}$  3:
**Action:**
  4:**For** each correlated region CR index *i* do  5:$CR_{D}^{i}$ = Density (One-hop neighbors of nanorouter in the correlated region *i*)  6:$CR_{E}^{i}$ = Energy (Residual energy in the correlated region *i*)  7:$CR_{PCMR}^{i}$ = Previous critical messages received (PCMR in the last data reporting interval in the correlated region *i*)  8:
**End For**
  9:Evaluate input parameters $\left(CR_{D}^{i},CR_{E}^{i},CR_{PCMR}^{i}\right)$ using Fuzzy Rules given in Figure 3. 10:**For** each $CR_{i}$ do 11:Calculate $Enrichment-value_{CR}$ (Using the defuzzification process). 12:Enriched list of CR(Enrichement({CR}) ← $Enrichment-value_{CR}$ 13:
**End For**
 14:
**Output:**
 15:$Enrichment-value_{CR}$ = List of Information and Energy enriched correlated regions

The fuzzy logic-based decision is made after completion of three main parts of the FLBDS, namely fuzzification, fuzzy inference system and defuzzification. Our proposed fuzzy logic-based decision model uses three input parameters (i.e., messages, density and energy) for generating one output parameter. The first input parameter gives knowledge about the number of messages that are received from a correlated region during the last interval. The second input parameter density shows the NBSs density at the one-hop distance from nanorouter and the last input parameter is energy that represents the remaining energy of the correlated region. Based on these three input parameters, the output parameter determines the enrichment value of a correlated region.

1. Fuzzification: In our proposed fuzzy logic-based decision model, the input and output parameters are described using the linguistic variables. The expressions used for input parameters, density, messages and energy are ‘high’, ‘medium’ and ‘low’, respectively. While the output parameter represents the enrichment value using the linguistic variables ‘very high’, ‘high’, ‘medium’, ‘low’ and ‘very low’. In the fuzzification process, these variables are represented by triangular membership functions, which are used to associate a grade to each input linguistic variable. The selection of the number of membership functions and their initial values is based on process knowledge and intuition. The membership function has a value between 0 and 1 over an interval of the crisp variable.
2. Fuzzy Inference System: After the fuzzification process, the fuzzified values are processed by the fuzzy inference engine to inference the fuzzy rules for driving decisions. The fuzzy rules used in our proposed fuzzy logic-based decision model are elaborated using Figure 3. The fuzzy rules are the sets of ‘IF’ and ‘THEN’ statements, for instance, the first rule is read as **IF** (Density is high) and (number of messages received are also high) and (energy is high) **THEN** (The enrichment of the correlated region is very high). The number of rules depends on the number of input membership functions, which grow exponentially with the increase in the number of input membership functions.
  The characteristics of the defined rules are imprecise; the value generated from the rules are fuzzy instead of crisp values. The input parameters are fuzzified according to the membership value to obtain crisp values. The attained crisp values are then used to determine the enrichment of the correlated-region.
3. Defuzzification: The obtained result is processed in the defuzzification process to achieve a quantifiable output (i.e., the enrichment of a correlated region). In this process, the membership degree of the fuzzy set is interpreted into some specific values (crisp value). In this work, the centroid (COG) method is used for defuzzification to obtain a crisp output value.

**Figure 4:**
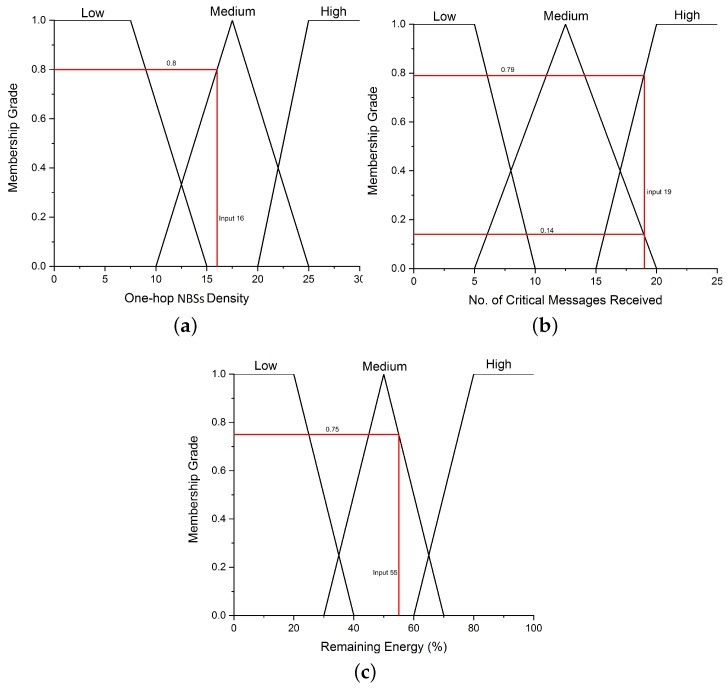
Input variables for performing fuzzification. (**a**) Input variable “density” for fuzzification. (**b**) Input variable “messages” for fuzzification. (**c**) Input variable “energy” for fuzzification.

**Example:** To further explain the fuzzy-based selection, we consider an example with three crisp inputs values; x = 16, y = 19 and z= 55 for density, messages and energy in the correlated regions, respectively as shown in Figure 4.

**Figure 5:**
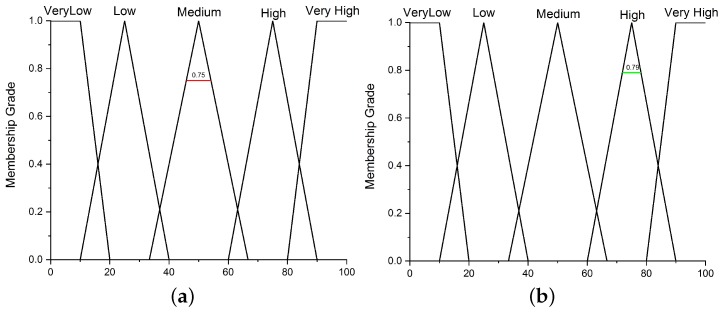
Mamdani Rule evaluation process for crisp input values of x = 17, y = 19 and z = 55. (**a**) If x is 0.8 (medium), y is 0.14 (medium) and z is 0.75 (medium) then the output w is 0.75 (medium). (**b**) If x is 0.8 (medium), y is 0.79 (high) and z is 0.75 (medium) then the output w is 0.79 (high).

After the fuzzification process, the fuzzified inputs are evaluated according to certain appropriate rules as shown in Figure 5. Based on the rules, each crisp value has a membership grade that is used for generating output. According to the rule, for the crisp input values (medium, medium and high), the output is medium. While for crisp input values (medium, high and medium), the output is high.

**Figure 6:**
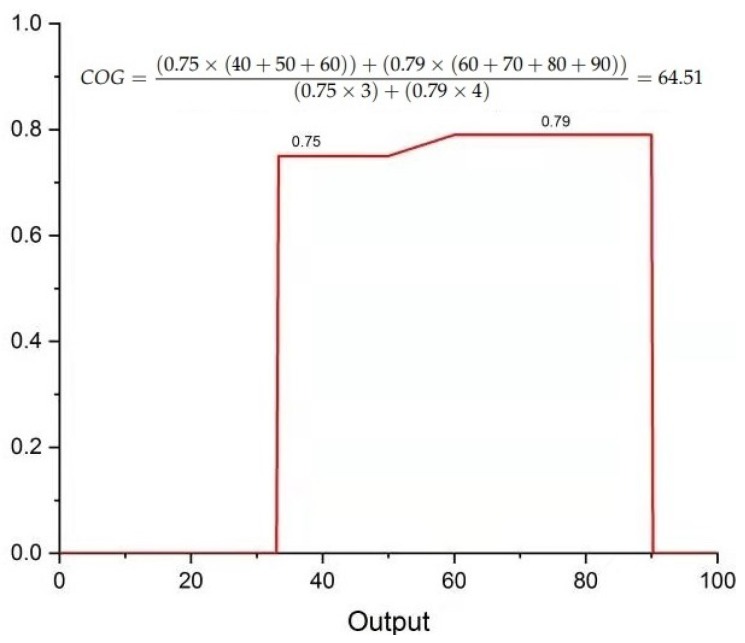
Mamdani defuzzification process using the centroid (COG) method for the crisp inputs values; x = 16, y = 19 and z = 55.

After completion of the Mamdani rule evaluation process, the defuzzification process is performed. In the defuzzification process, the attained aggregated fuzzy set is converted to a number through the COG technique as demonstrated in Figure 6.

### 3.3. Bio-Inspired Firefly Algorithm Based NBSs Selection

The firefly algorithm is inspired by the flashing pattern and behavior of fireflies. The low-complexity and self-adaptability of this metaheuristic approach make it the most suitable approach for adaptation in IBNNs for designing low-complexity routing schemes. Fireflies use their flashlight for attracting other fireflies for communication and mating; the flashing pattern, rhythm and the rate influence the attractiveness of a firefly. The firefly algorithm is based on the three main assumptions; first, all the fireflies are unisex. Second, the attractiveness of a firefly is proportional to its intensity, as the distance increases both the attractiveness and intensity decreases. If no brighter firefly is found then the particular firefly moves randomly. Third, the objective function determines and influences the intensity of the firefly. The attractiveness of firefly is determined using Equation (5):

(5) $$\zeta \left(d\right)=\zeta_{o}e^{-\beta d^{2}}$$

where ζ(d) expresses the attractiveness of a firefly at a distance *d*. The attractiveness of a firefly at a distance d=0 is represented as $\zeta_{o}$. β and *d* denotes light absorption coefficient and distance between two fireflies respectively. The distance between two fireflies ${\tilde{FF}}_{i}$ and ${\tilde{FF}}_{j}$ is calculated using Equation (6):

(6) $$d\left({\tilde{FF}}_{i},{\tilde{FF}}_{j}\right)=\sqrt{{\left({\tilde{FF}}_{j}\left(x\right)-{\tilde{FF}}_{i}\left(x\right)\right)}^{2}+{\left({\tilde{FF}}_{j}\left(y\right)-{\tilde{FF}}_{i}\left(y\right)\right)}^{2}+{\left({\tilde{FF}}_{j}\left(z\right)-{\tilde{FF}}_{j}\left(z\right)\right)}^{2}},$$

where $d({\tilde{FF}}_{i},{\tilde{FF}}_{j})$ is the distance from the coordinates of firefly ${\tilde{FF}}_{i}$ given by $\left({\tilde{FF}}_{i}\left(x\right),{\tilde{FF}}_{i}\left(y\right),{\tilde{FF}}_{i}\left(z\right)\right)$ to the coordinates of firefly ${\tilde{FF}}_{j}$ denoted as $\left({\tilde{FF}}_{j}\left(x\right),{\tilde{FF}}_{j}\left(y\right),{\tilde{FF}}_{j}\left(z\right)\right)$.

Since all the spatially correlated NBSs are assumed to detect similar types of information, therefore, limiting the participation of NBSs in the data reporting process increases the network lifetime by conserving network resources that are consumed in redundant data transmission. The proposed firefly algorithm optimizes NBSs selection for reducing redundant data transmission, balancing energy consumption and increasing the probability of retrieving more valuable information from NBSs. The proposed firefly algorithm for the optimal selection of NBSs is further provided in Algorithm 2. The detailed demonstration of the main steps of our proposed firefly algorithm is given below:

1. Solution Initialization: In the firefly algorithm, the potential solution of a problem is represented by each firefly. Similarly, in NBSs selection, each firefly symbolizes as the solution of NBSs selection problem. The proposed algorithm first initializes the initial solution. The initial solution structure is generated randomly (i.e., 0 or 1) depending on the number of NBSs.
2. Fitness function calculation: In the view of the influence of the light intensity on the brightness of a firefly, in this work, the attractiveness of the firefly devise its fitness. The fitness value is based on the three main characteristics; remaining energy of the NBSs, distance from the nanorouter and the priority bit.
  (a) Residual Energy: Selecting NBSs with more residual energy assist in prolonging the network lifetime. Therefore, selecting NBSs with more energy available is a more preferable choice.
  (b) Distance: Data transmission at less distance requires low energy consumption and ultimately increased network lifetime. Therefore, NBSs that is located at the minimum distance from nanorouter has better fitness value.
  (c) Priority Bit: The priority bit depends on the content of the last transmitted data, if the NBS has transmitted critical messages in the last interval then priority bit is set to 1 otherwise it is 0.
  Based on the fitness parameters, the overall fitness value of a NBS is determined using Equation (7):
  (7) $$NBS_{i}\left(Fitness\right)=\alpha_{1}\times E_{i}+\alpha_{2}\times \frac{1}{d(i,r)}+\alpha_{3}\left(Prioritybit\right),$$
  where weights α are used to balance the weight of three parameters expressed as $\alpha_{i}\in \left(0,1\right)$ and $\alpha_{1}+\alpha_{2}+\alpha_{3}=1$.
3. New Solution Updating: After determining the light intensity of each solution, the firefly with less brightens moves to the next firefly with more brightness. The position of the next firefly is updated using the Equation (8):
  (8) $${\tilde{FF}}_{i}\left(pos+1\right)={\tilde{FF}}_{i}\left(pos\right)+\zeta_{o}^{-\beta d_{\tilde{FFi},\tilde{FFj}}^{2}}\left({\tilde{FF}}_{j}\left(x\right),{\tilde{FF}}_{i}\left(x\right)\right)+\alpha \left(random-1/2\right),$$
  where ${\tilde{FF}}_{i}$ and ${\tilde{FF}}_{j}$ are two fireflies, α denoted the randomization parameter and random function is used to generate random value between [0,1].

**Algorithm 2:** Firefly algorithm based NBSs selection  1:**Initialize population**$nbs=(nbs_{1},nbs_{2},\ldots nbs_{i})T$  2:Perform the generation of initial firefly population, (1,2,…i)  3:Calculate the light intensity *I* at $nbs_{i}$ using Equation (7) and obtain the $I_{best}$.  4:**While** t < MAXGeneration  5:**For** every i  6:**For** every j  7:**update** fitness function and find light intensity.  8:**For**$n_{f}=1:N_{flies}$  9:**If**$max(I_{nf}$ > $I^{best})$
*then* 10:**Replace** with updated firefly. 11:**Else** Randomly change function and generate new intensity. 12:**End If**13:**End For** 14:Determine the new solution using Equation (8) and update the light intensity. 15:**End For** 16:**End For** 17:Make a ranking of the fireflies to locate the present best. 18:**End While**

### 3.4. Data Transmission

Once the selected NBSs receive announcement message $P_{AM}$, NPSs transmit the response messages according to the assigned transmission status. The status is allocated to the NSBs according to the following Equation (9):

(9) $$NBS_{i}\left(status\right)=\frac{EV_{CR(r)}+FV_{NBS(i)}}{TotalNo.ofselectedNBSs},$$

where $EV_{CR(r)}$ is the enrichment value of the correlated region indexed *r* and $FV_{NBS(i)}$ is the fitness value of the NBS indexed *i*.

### 3.5. The Lifetime of an IBNN

Network lifetime is the fundamental characteristic of an IBNN for evaluating its potential in specific applications. In the existing literature, various factors are considered, such as the alive number of nodes, sensor coverage and connectivity for defining the lifetime of a WSN [59]. Similarly, the lifetime of IBNN is also associated with the number of alive NBSs, defined as the time when NBSs exhaust themselves and have no energy left for carrying out primary operations. The definition provides information that when NBSs do not have enough energy to transmit data packets, the network can no longer provide useful information to the external healthcare system. Therefore, the IBNN should be considered dead. The evaluation of IBNN lifetime based on the alive number of NBSs is briefly discussed in this section.

The detailed comprehension of IBNN lifetime is possible by briefly explaining the life cycle of an NBS. The life span of an NBS depends on the number of messages transmitted and received by the nano-transceivers. Nano-transceivers use TS-OOK modulation scheme for communication in the THz band as THz band transceivers are not able to handle the shape or phase complexity of the transmitted signal [60]. At the physical layer, TS-OOK modulation scheme is used with pulse duration, pulse time interval and transmission range equal to 100 fs, 100 ps and 10 mm, respectively. According to TS-OOK modulation scheme the energy consumed by a NBS for transmitting a pulse is 1 pJ (i.e., $E_{tx}$ = 1 pJ) and the amount of energy needs for a pulse reception is 0.1 pJ (i.e., $E_{rx}$ = 1 pJ). Thus, the energy consumed by the *i*th NBS in the transmission and reception of *x* number of bits is given as Equations (10) and (11):

(10) $$E_{tx}\left(x\right)=x\times \alpha \times E_{tx}^{pulse}$$

(11) $$E_{rx}\left(x\right)=0.1\times x\times E_{rx}^{pulse},$$

where weight α is used in Equation (10) to denote the probability of transmitting the symbol 1 in the *x* streams of bits. Since the symbols 0 and 1 are equiprobable, therefore, the value of α is generally set to 0.5. While in the case of energy consumption during the reception, the entire *x* stream of bits is considered. Accordingly, the total energy consumed by the *i*th NBS in the *r*th round expressed as Equation (12):

(12) $$E\left(nbs_{i}^{r}\right)=\left(P_{RM}\times E_{tx}+P_{FB}\times E_{tx}+P_{RqM}\times E_{rx}+P_{AM}\times E_{rx}\right),$$

where $P_{RM}$, $P_{FB}$, $P_{RqM}$ and $P_{AM}$ shows the total number of transmitted and received packets respectively. While $E_{tx}$ and $E_{rx}$ represents the amount of energy consumed in packet transmission and reception, respectively. Consequently, the amount of energy left in the NBS after completion of *r*th round is determined using Equation (13):

(13) $$RE\left(nbs_{i}^{r}\right)=E\left(nbs_{i}^{r}\right)+E_{h}^{r},$$

where $E(NBS_{i}^{r})$ is the energy consumption of the *i*th NBS and $E_{h}^{r}$ is the amount of energy harvested in the *r*th round.

In the context of energy harvesting mechanisms in nanonetworks, conventional mechanisms such as solar energy, wind power, or underwater turbulence are inapplicable due to the technical limitations of these schemes at NS level. In the last few years, novel schemes are introduced for recharging NS devices. Piezoelectric nano-generator, which is composed of an array of ZnO nanowires, a reflecting circuit and an ultra nanocapacitor, has gained significant attention for recharging NS devices. The compression of nano-wires generates an electric current between the ends of nanowires that are used for recharging the ultra nanocapacitor. The mechanical vibration methods (such as air conditioning and heartbeat) provides the compressed release cycle. The frequency of the compressed release cycles *f* provides the time for recharging ultra nano-capacitor [61]. In IBNNs, NBSs are integrated inside the human body and the only possible source of mechanical vibration inside the human body is a heartbeat, therefore, f=1 Hz.

According to the energy harvesting model, after α compressed cycles, the voltage of the charging capacitor is calculated using Equation (14):

(14) $$V_{cap}\left(\alpha_{cycles}\right)=V_{g}\times \left(1-e^{-\frac{\alpha_{cycles}\Delta Q}{V_{g}C_{cap}}}\right),$$

where $C_{cap}$ is the total capacitance of the ultra-nanocapacitor, ΔQ represents the harvested charge per cycle and $V_{g}$ is the generator voltage. However, due to technical limitation of NBSs, the typical values of quantities $C_{cap}$, ΔQ and $V_{g}$ are 9 cF, 6 pc and 0.42 V respectively. The energy $E_{cap}$ stored in ultra-nanocapacitor after $\alpha_{cycles}$ is re-written as [61] using Equation (15):

(15) $$E_{cap}\left(\alpha \right)=\frac{1}{2}C_{cap}\left(V_{g}{\left(\alpha_{cycles}\right)}^{2}\right),$$

where $C_{cap}$ corresponds to entire capacitance of ultra-nanocapacitor, $V_{g}$ signifies the voltage of the generator and $\alpha_{cycles}$ demonstrates the number of cycles necessary to reach the energy level given by Equation (16):

(16) $$\alpha_{cycles}\left(E\right)=\lceil \frac{-V_{g}C_{cap}}{\Delta Q}ln(1-\sqrt{\frac{2E}{C_{cap}V_{g}^{2}}})\rceil ,$$

where $V_{g}$ is the generator voltage, $C_{cap}$ is the capacitance of ultra-nanocapacitor and ΔQ represents the harvested charge per cycle. Following the above given information, the energy harvested by the *i*th NBS in the *r*th round is obtained by determining the number of cycles α required for charging the ultra-nanocapacitor, as expressed in Equation (17):

(17) $$E_{h}^{r}\left(i\right)=E_{cap}\left(cycles_{\alpha }+cycles_{r}\right),$$

where $cycles_{\alpha }$ refers to number of cycles given in Equation (16) and $cycles_{r}$ represents number of cycles in the *r*th interval.

Considering the amount of energy harvested and consumed in packet transmission and reception, the lifetime of the *i*th NBS is obtained using Equation (18):

(18) $$LifeTime\left(nbs^{i}\right)=\frac{E_{o}^{i}}{RE(nbs_{i}^{r})},$$

where $E_{o}^{i}$ is the initial energy of the NBS and $RE(NBS_{i}^{r})$ is the remaining energy of the NBS. Consequently, the lifetime of an IBNN is defined using Equation (19):

(19) $$LifeTime_{NW}=min\left\{L_{m}|\overset{︷}{NBS_{D}},L_{|\overset{︷}{NBS_{ND}}|}^{k},|\overset{︷}{NBS_{ND}}|\right\}.$$

## 4. Performance Evaluation

This section aims at evaluating the proposed protocol for validating the practical utility of the proposed protocol in the biomedical field, by studying a health-monitoring system based on IBNN. The evaluation is performed based on the residual energy, network lifetime, average end-to-end delay and packet delivery ratio. We have compared our proposed scheme with the selective flooding scheme, which is considered as the benchmark for evaluating electromagnetic-based IBNNs [19]. Moreover, intending to prove an in-depth evaluation of our proposed scheme, we have also compared our scheme with different scenarios. In the first scenario, all the correlated regions are involved in the data aggregation process (i.e., without employing fuzzy logic-based correlated region selection) and only NBSs are optimally selected using bio-inspired firefly algorithm. While in the second scenario, only the correlated regions are selected and all the NBSs located in the selected correlated regions generate response messages $\left(P_{RM}\right)$. We referred to both scenarios as scenarios A and B in the result section for simplicity. The simulations are run ten times to obtain the results demonstrated in the result sections. The detailed comparison assists in investigating the potential of proposed work and forecasts future research.

### 4.1. Nano-SIM Simulation

The proposed protocol is simulated using the Nano-SIM tool, Nano-SIM tool is an emerging tool integrated into NS3 for simulating the performance of electromagnetic-based nanonetworks. In line with Reference [17], the area of the considered IBNN is a 10 mm radius. The considered IBNN consists of (100 and 200) NBSs and one nanorouter, where NBSs are moving using constant mobility model and nanorouter maintains a fixed position at the center of the network area. All NBSs are equipped with a sensing unit and are able to sense the surrounding environment and to collect information about chemical particles and biological functions. Nanorouter periodically collects data from NBSs to update the external healthcare system about the health condition. The requests are generated in intervals in the range of [0.2–0.5] requests/s. The physical layer is set up according to Reference [60]. The size of request messages and feedback messages is 48 bits, while the size of response messages flowing in the network is 176 bits, respectively. To provide a fair comparison, we have used the same simulation parameter settings for flooding scheme and other comparison scenarios. The simulation settings are further highlighted in the Table 2.

#### 4.1.1. Residual Energy

Residual energy is defined as the energy left in the network at the end of the simulation. We have evaluated remaining energy with respect to different request intervals to comprehend the influences of request rate on the energy resources of the network. In this work, we evaluate residual energy with different request rate intervals and increasing densities of NBSs.

#### Residual Energy in the Network with the Density of 100 NBSs and Increasing Request Rate Intervals

**Figure 7:**
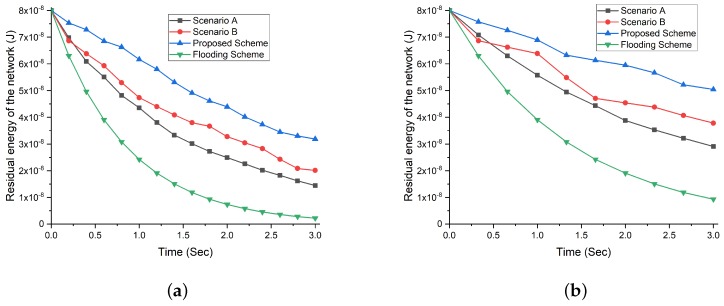

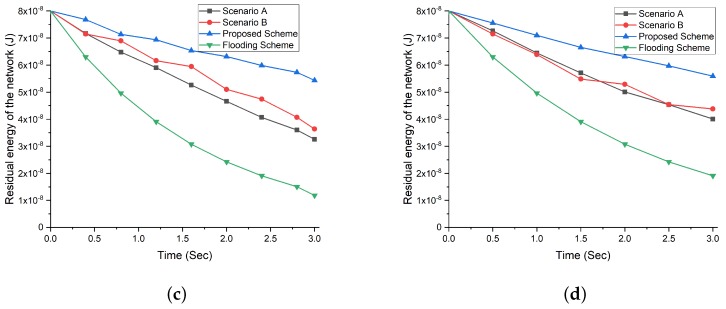
Residual energy comparison of our proposed scheme with flooding scheme and different scenarios (A and B) with respect to increasing request rate interval and NBSs density of 100. (**a**) Residual energy comparison at request rate interval 0.2. (**b**) Residual energy comparison at request rate interval 0.3. (**c**) Residual energy comparison at request rate interval 0.4. (**d**) Residual energy comparison at request rate interval 0.5.

Figure 7 demonstrates that the increasing rate of request generation leads to a fast depletion of energy resources. For the request rate interval 0.2, the maximum number of requests are generated in the network and to satisfy a large number of requests, a large number of answer messages are generated that cause high energy consumption. The detailed comparison in Figure 7a–d depict that employing a fuzzy logic and bio-inspired firefly algorithm in our proposed scheme maximum amount of energy is left at all intervals (i.e., 0.2,0.3,0.4 and 0.5). The reason behind the highest residual energy is due to data aggregation prevention from low enrichment value correlated region and optimum selection of NBSs with the highest fitness value that supports decreased energy consumption. While in scenarios A and B, the remaining energy is almost the same due to the selection of the optimal number of NBSs and information and energy enriched correlated regions. Both scenarios provide better remaining energy than the flooding scheme as in the flooding scheme, increased the number of packet generation and forwarding rate results in consuming all the energy in the network before the simulation ends.

#### Residual Energy in the Network with the Density of 200 NBSs and Increasing Request Rate Intervals

**Figure 8:**
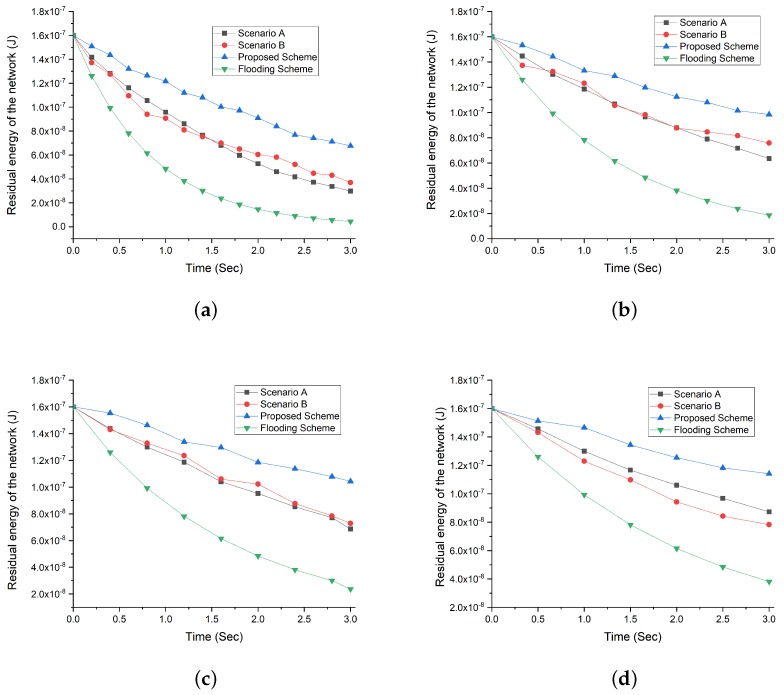
Residual energy comparison of our proposed scheme with flooding scheme and different scenarios (A and B) with respect to increasing request rate interval and NBSs density of 200. (**a**) Residual energy comparison at request rate interval 0.2. (**b**) Residual energy comparison at request rate interval 0.3. (**c**) Residual energy comparison at request rate interval 0.4. (**d**) Residual energy comparison at request rate interval 0.5.

The increase in the number of NBSs increases the probability of having active NBSs in the network. However, the rise in the request rate interval increases the number of packet transmission and reception rate. The Figure 8 shows that flooding scheme consumes its available energy resources at the highest rate at all intervals (see Figure 8a–d ). While our proposed scheme achieves the best result for an increasing number of NBSs due to the optimum selection of correlated region and NBSs. The scenario A and B, both witness high energy consumption as compared to our proposed scheme as selecting only the correlated region or only selection of optimum NBSs cannot solely overcome the vast consumption of energy resources.

#### 4.1.2. Network Lifetime

Network lifetime is defined based on the number of alive NBSs (m-ink-of-n nodes) at the end of simulations. The number of alive NBSs are evaluated with respect to different request rate intervals and with increasing density of NBSs in the cluster.

#### Network Lifetime with the Density of 100 NBSs and Increasing Request Rate Intervals

**Figure 9:**
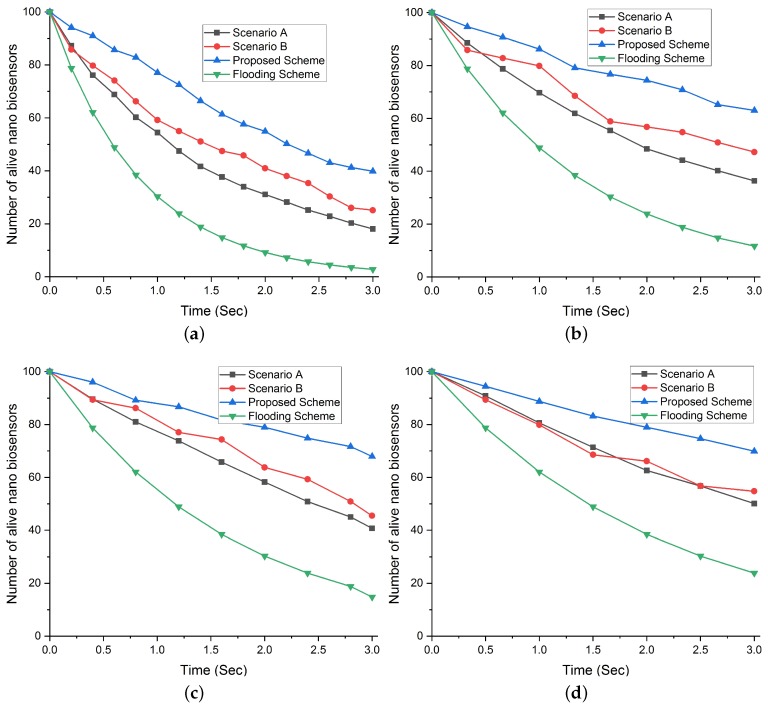
Comparison of total number of alive NBSs in our proposed scheme with flooding scheme and different scenarios (A and B) with respect to increasing request rate interval and NBSs density of 100. (**a**) Total number of alive NBSs at request rate interval 0.2. (**b**) Total number of alive NBSs at request rate interval 0.3. (**c**) Total number of alive NBSs at request rate interval 0.4. (**d**) Total number of alive NBSs at request rate interval 0.5.

Figure 9 clearly depicts that the maximum number of alive NBSs are obtained for the 0.5 request rate interval. Since at interval 0.5, requests are generated at the minimum number. Our proposed scheme achieves the maximum network lifetime due to our selection strategy that balances the message generation load among all the NBSs. In addition, the selection of NBSs based on the their fitness value also supports in preventing energy consumption in those NBSs that have low remaining energy. While Flooding scheme results in a minimum network lifetime for all the intervals due to the involvement of all the NBSs. From Figure 9a–d, it is evident that scenarios A and B do not provide improved network lifetime alone, without combining them to achieve the goal of maximum network lifetime.

#### Network Lifetime with the Density of 200 NBSs and Increasing Request Rate Intervals

**Figure 10:**
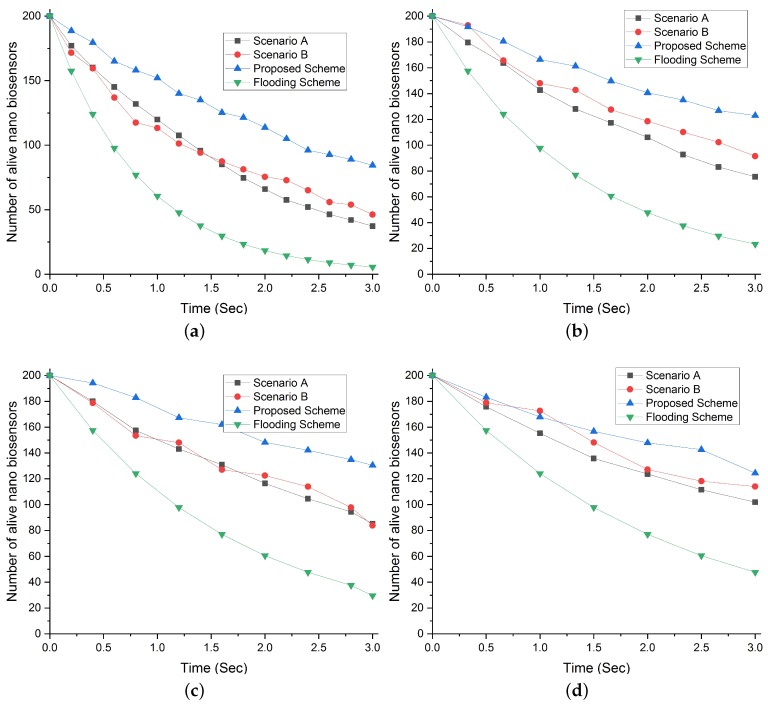
Comparison of total number of alive NBSs in our proposed scheme with flooding scheme and different scenarios (A and B) with respect to increasing request rate interval and NBSs density of 200. (**a**) Total number of alive NBSs at request rate interval 0.2. (**b**) Total number of alive NBSs at request rate interval 0.3. (**c**) Total number of alive NBSs at request rate interval 0.4. (**d**) Total number of alive NBSs at request rate interval 0.5.

From Figure 10, it is evident that increasing the density of NBSs improves network lifetime. However, the increasing density also influences the rate of packet generation. In our proposed scheme, the network lifetime remains almost the same for increasing the density of NBSs due to our proposed selection scheme. While in both scenarios A and B, the number of dead NBSs increases with the increase in the request rate interval. Similarly, the flooding scheme experienced the minimum network lifetime for all the intervals.

#### 4.1.3. Average Remaining Energy Comparison at the End of Simulations with Increasing Request Rate Interval and NBSs Density

**Figure 11:**
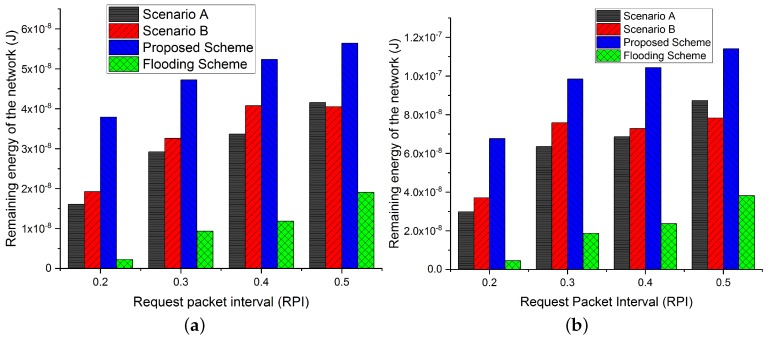
Total remaining energy in the network comparison of proposed scheme with flooding scheme and different scenarios (A and B) with respect to increasing request rate interval and NBSs density of 100 and 200. (**a**) Total remaining energy in the network with respect to increasing request rate interval and NBSs density of 100. (**b**) Total remaining energy in the network with respect to increasing request rate interval and NBSs density of 200.

The remaining energy at the end of the simulation with different NBSs density is represented in Figure 11. The Figure shows that our proposed scheme outperforms all the other scenarios and flooding scheme for both densities of NBSs. By combing the characteristics of fuzzy logic and bio-inspired firefly algorithm, our proposed scheme results in the highest remaining energy. While in both scenarios A and B, the remaining energy is almost similar due to the optimal selection of correlated regions and NBSs. Flooding scheme has the lowest remaining energy due to the participation of all the NBSs in data reporting, which also significantly increases the overall packet forwarding rate. Therefore, the flooding scheme consumes more energy.

#### 4.1.4. Average End-to-End Delay

**Figure 12:**
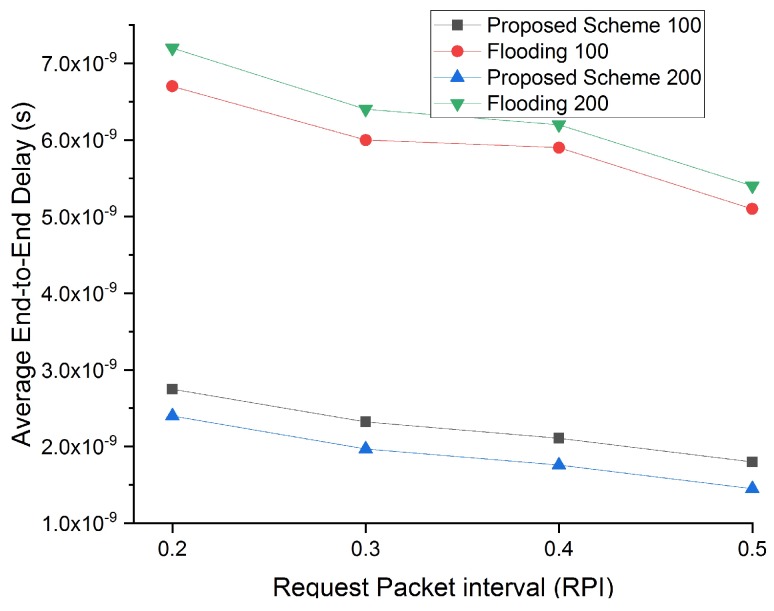
Average end-to-end delay comparison with flooding scheme with respect to increasing request rate intervals and NBSs density of 100 and 200.

Average end-to-end delay is defined as the time a packet takes from its transmission till the reception. The average end-to-end delay is compared for different request rate intervals and increasing NBSs density to provide meaningful insights into our proposed work Figure 12.

Figure 12 clearly shows that our proposed scheme experiences the lowest average end-to-end delay at all intervals with NBSs density of 100 and 200 in a cluster. Our proposed scheme of selecting correlated regions and an optimal number of NBSs avoids the transmission of redundant data. Therefore, a lower number of packets awaits in the queue for their turn in the transmission, which ultimately reduces the average end-to-end delay experienced by a packet. Further, NBSs fitness value also considers the distance of NBSs from nanorouter for reducing packet forwarding rate. Avoiding redundant data transmission and controlling packet forwarding rate results in low average end-to-end delay even for increased nanosensor density. While in the flooding scheme, the number of packet transmission and packet forwarding is higher, that significantly increases the average end-to-end delay for both densities of NBSs.

#### 4.1.5. Packet Delivery Ratio

The successful reception of the maximum number of packets leads to increased packet delivery ratio. The packet delivery ratio is assessed with respect to different request rate intervals to highlight the impact of request rate intervals on the packet delivery ratio.

**Figure 13:**
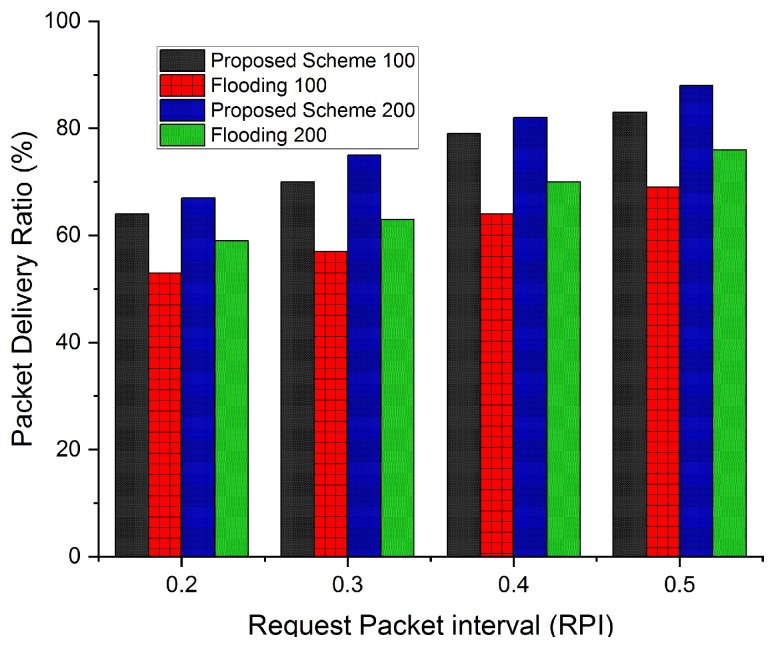
Packet delivery ratio comparison with flooding scheme with respect to increasing request rate intervals and NBSs density of 100 and 200.

Selecting correlated regions and NBSs also improve the packet delivery ratio, as shown in Figure 13. When the number of packets generated by NBSs are controlled, it also reduces congestion and packet drop ratio. Therefore, our proposed scheme achieves a better packet delivery ratio as compared to the flooding scheme. In the flooding scheme, all the NBSs generate response messages at fixed intervals that increase the rate of packets flowing in the network. Due to the smaller range of NBSs, packets are also forwarded at a higher rate. The higher packet transmission and packet forwarding rate in the flooding scheme, consequently result in lower packet delivery ratio for both densities of NBSs.

## 5. Conclusions

In this work, a novel energy-efficient routing protocol is proposed that explicitly considers the constraints of NBSs in providing continuous healthcare, diagnostics and treatments. In our presented work, we have proposed the FLBDS and bio-inspired firefly algorithm for deciding on the selection of correlated regions and NBSs. The proposed routing scheme devises a new scheme that exchanges the burden of generating periodic response among all the members of IBNNs. Furthermore, our proposed scheme also prevents redundant data transmission and increases the transmission of crucial information. The proposed FLBDS ensures the selection of only those correlated regions that have valuable information and enough residual energy resources to handle the data communication load. While the selection of NBSs based on our new fitness function permits only those NBSs participation in data reporting that have maximum residual energy and have already transmitted crucial information in the last interval. Simulation results validate that the proposed scheme outperforms flooding scheme and the diverse scenarios opted for comparison and offer better residual energy, network lifetime, average end-to-end delay and packet delivery ratio. From the results, we conclude that our proposed scheme has a higher potential for realizing energy-efficient routing in IBNNs for more realistic healthcare applications.

## Figures and Tables

**Table 1:** The impact of employing a Fuzzy Logic-Based Decision System (FLBDS) in improving performance of Wireless Sensor Networks (WSNs).

Protocol Name	Purpose of Using FLBDS	Impact on Performance
Fuzzy-Logic-Based Energy Optimized Routing for WSNs [23].	Optimize next-hop selection considering the energy of the next-hop and closeness to the shortest path and the sink.	Extended network lifetime, energy efficiency and energy balance.
Cluster-head Election using Fuzzy Logic for WSNs [24].	Selection of cluster heads on the basis of energy and concentration.	A significant increase in network lifetime.
Multi-Sensor Data Fusion in Cluster-based WSNs Using Fuzzy Logic Method [25].	Data processing and fusion is carried out using fuzzy rule-based system. Cluster heads process collected information using the fuzzy rule-based system.	Reduce false alarm rate by improving the reliability and accuracy of the sensed information.
A Fuzzy Logic-Based Clustering Algorithm for WSN to Extend the Network Lifetime [26].	Appropriate cluster head selection based on residual energy, base station mobility and cluster centrality.	Improved network lifetime and stability.
Cluster Head Selection in WSNs under Fuzzy Environment [27].	Cluster head selection on the basis of residual energy, neighbors density, and the distance.	Prolonged network lifetime.
Swarm intelligence based fuzzy routing protocol for clustered WSNs [28].	Balanced clustering of nodes and efficient selection of cluster head based on residual energy, distance, and cluster centroid.	Support heterogeneous applications, improved network lifetime and balanced cluster generation.
Improving on LEACH Protocol of WSNs Using Fuzzy Logic [29].	Sink calculates the chance of each node to become a cluster head.	Make a better selection of cluster head for energy saving.
CHEF: Cluster Head Election mechanism using Fuzzy logic in WSNs [30].	Optimize cluster head selection based on the residual energy and local distance.	Prolonged network lifetime
Fuzzy-Logic-Based Clustering Approach for WSNs Using Energy Predication [31].	Improved cluster head selection on the basis of its residual energy and expected energy for the next round.	Energy efficiency
Energy Efficient Cross-Layer Routing Protocol in WSNs Based on Fuzzy Logic [32].	Fuzzy logic-based next-hop routing decision.	Maximize network lifetime
A clustering routing protocol for WSNs based on type-2 fuzzy logic and ACO [33].	Fuzzy logic-based cluster head selection based on residual energy, neighbor nodes density and distance.	Improved load balancing and network lifetime
An energy-aware fuzzy approach to unequal clustering in WSNs [34].	Using fuzzy logic approach to handle uncertainties in cluster-head radius estimation.	Energy-efficient data aggregation
MOFCA: Multi-objective fuzzy clustering algorithm for WSNs [35].	Energy-based fuzzy competition is carried out for cluster head nomination.	Prolonged network lifetime

**Table 2:** The values of parameters selected for the simulations.

Parameter	Value
Number of NBSs	100, 200
Number of nanorouter	1
TTL value	100
Tx Range of NBS (mm)	10
Request packet interval	0.2, 0.3, 0.4, 0.5
Pulse energy (pJ)	100
Pulse duration (fs)	100
Pulse interval time (ps)	10
Packet size $\left(P_{FB},P_{RqM}\right)$	48 (bits)
Packet size $\left(P_{RM}\right)$	176 (bits)
Simulation duration (s)	3
Total iteration	10

## Abbreviations

The following abbreviations are used in this manuscript:

IBNN	Intrabody Nanonetwork
NBS	Nano Biosensors
NS	NanoScale
FLBDS	Fuzzy Logic-Based Decision System
WSN	Wireless Sensor Network
THz	Terahertz Band

## References

[1] Chen M., Gonzalez S., Vasilakos A., Cao H., Leung V.C. (2011). Body area networks: A survey. Mob. Netw. Appl..

[2] Cao H., Leung V., Chow C., Chan H. (2009). Enabling technologies for wireless body area networks: A survey and outlook. IEEE Commun. Mag..

[3] Palejwala A.H., Fridley J.S., Mata J.A., Samuel E.L., Luerssen T.G., Perlaky L., Kent T.A., Tour J.M., Jea A. (2016). Biocompatibility of reduced graphene oxide nanoscaffolds following acute spinal cord injury in rats. Surg. Neurol. Int..

[4] Jokerst J.V., Raamanathan A., Christodoulides N., Floriano P.N., Pollard A.A., Simmons G.W., Wong J., Gage C., Furmaga W.B., Redding S.W. (2009). Nano-bio-chips for high performance multiplexed protein detection: determinations of cancer biomarkers in serum and saliva using quantum dot bioconjugate labels. Biosens. Bioelectron..

[5] Choi Y.E., Kwak J.W., Park J.W. (2010). Nanotechnology for early cancer detection. Sensors.

[6] Wu L., Qu X. (2015). Cancer biomarker detection: Recent achievements and challenges. Chem. Soc. Rev..

[7] Huang Y.F., Shangguan D., Liu H., Phillips J.A., Zhang X., Chen Y., Tan W. (2009). Molecular assembly of an aptamer–drug conjugate for targeted drug delivery to tumor cells. ChemBioChem.

[8] Jain K.K. (2007). Applications of nanobiotechnology in clinical diagnostics. Clin. Chem..

[9] Akyildiz I.F., Brunetti F., Blázquez C. (2008). Nanonetworks: A new communication paradigm. Comput. Netw..

[10] Akyildiz I.F., Jornet J.M., Han C. (2014). Terahertz band: Next frontier for wireless communications. Phys. Commun..

[11] Akyildiz I.F., Jornet J.M., Pierobon M. (2011). Nanonetworks: A new frontier in communications. Commun. ACM.

[12] Llatser I., Kremers C., Cabellos-Aparicio A., Jornet J.M., Alarcón E., Chigrin D.N. (2012). Graphene-based nano-patch antenna for terahertz radiation. Photonics NanoStruct. Fundam. Appl..

[13] Piro G., Yang K., Boggia G., Chopra N., Grieco L.A., Alomainy A. (2015). Terahertz communications in human tissues at the nanoscale for healthcare applications. IEEE Trans. Nanotechnol..

[14] Jornet J.M., Akyildiz I.F. (2011). Channel modeling and capacity analysis for electromagnetic wireless nanonetworks in the terahertz band. IEEE Trans. Wirel. Commun..

[15] Piro G., Boggia G., Grieco L.A. (2015). On the design of an energy-harvesting protocol stack for Body Area Nano-NETworks. Nano Commun. Netw..

[16] Liu J., Wu Z.X. (2017). On the design of an energy-efficient data collection scheme for body area nanonetworks. Int. J. Wirel. Mob. Netw. (IJWMN).

[17] Afsana F., Asif-Ur-Rahman M., Ahmed M.R., Mahmud M., Kaiser M.S. (2018). An energy conserving routing scheme for wireless body sensor nanonetwork communication. IEEE Access.

[18] Lee S.J., Jung C., Choi K., Kim S. (2015). Design of wireless nanosensor networks for intrabody application. Int. J. Distrib. Sens. Netw..

[19] Piro G., Grieco L.A., Boggia G., Camarda P. Nano-Sim: Simulating electromagnetic-based nanonetworks in the network simulator 3. Proceedings of the 6th International ICST Conference on Simulation Tools and Techniques.

[20] Zadeh L.A. (1965). Fuzzy sets. Inf. Control.

[21] Klir G.J., Yuan B. (1995). Fuzzy Sets and Fuzzy Logic: Theory and Applications.

[22] Tzeng G.H., Huang J.J. (2016). Fuzzy Multiple Objective Decision Making.

[23] Jiang H., Sun Y., Sun R., Xu H. (2013). Fuzzy-logic-based energy optimized routing for wireless sensor networks. Int. J. Distrib. Sens. Netw..

[24] Gupta I., Riordan D., Sampalli S. Cluster-head election using fuzzy logic for wireless sensor networks. Proceedings of the 3rd Annual Communication Networks and Services Research Conference (CNSR’05).

[25] Manjunatha P., Verma A., Srividya A. Multi-sensor data fusion in cluster based wireless sensor networks using fuzzy logic method. Proceedings of the 2008 IEEE Region 10 and the Third International Conference on Industrial and Information Systems.

[26] Nayak P., Devulapalli A. (2015). A fuzzy logic-based clustering algorithm for WSN to extend the network lifetime. IEEE Sens. J..

[27] Azad P., Sharma V. (2013). Cluster head selection in wireless sensor networks under fuzzy environment. ISRN Sens. Netw..

[28] Zahedi Z.M., Akbari R., Shokouhifar M., Safaei F., Jalali A. (2016). Swarm intelligence based fuzzy routing protocol for clustered wireless sensor networks. Expert Syst. Appl..

[29] Ran G., Zhang H., Gong S. (2010). Improving on LEACH protocol of wireless sensor networks using fuzzy logic. J. Inf. Comput. Sci..

[30] Kim J.M., Park S.H., Han Y.J., Chung T.M. CHEF: Cluster head election mechanism using fuzzy logic in wireless sensor networks. Proceedings of the 2008 IEEE 10th International Conference on Advanced Communication Technology.

[31] Lee J.S., Cheng W.L. (2012). Fuzzy-logic-based clustering approach for wireless sensor networks using energy predication. IEEE Sens. J..

[32] Jaradat T., Benhaddou D., Balakrishnan M., Al-Fuqaha A. Energy efficient cross-layer routing protocol in wireless sensor networks based on fuzzy logic. Proceedings of the 2013 IEEE 9th International Wireless Communications and Mobile Computing Conference (IWCMC).

[33] Zhang Q.Y., Sun Z.M., Zhang F. A clustering routing protocol for wireless sensor networks based on type-2 fuzzy logic and ACO. Proceedings of the 2014 IEEE international conference on fuzzy systems (FUZZ-IEEE).

[34] Bagci H., Yazici A. (2013). An energy aware fuzzy approach to unequal clustering in wireless sensor networks. Appl. Soft Comput..

[35] Sert S.A., Bagci H., Yazici A. (2015). MOFCA: Multi-objective fuzzy clustering algorithm for wireless sensor networks. Appl. Soft Comput..

[36] Eberhart R., Kennedy J. A new optimizer using particle swarm theory. Proceedings of the Sixth International Symposium on Micro Machine and Human Science MHS’95.

[37] Dorigo M., Birattari M. (2010). Ant Colony Optimization.

[38] Yang X.S. Firefly algorithms for multimodal optimization. Proceedings of the International Symposium on Stochastic Algorithms.

[39] Pavai K., Sivagami A., Sridharan Study of routing protocols in wireless sensor networks. Proceedings of the 2009 IEEE International Conference on Advances in Computing, Control, and Telecommunication Technologies.

[40] Dressler F. (2006). Benefits of bio-inspired technologies for networked embedded systems: An overview. Dagstuhl Seminar Proceedings.

[41] Bitam S., Mellouk A. (2014). Vehicular ad hoc networks. Bio-Inspired Routing Protocols for Vehicular Ad Hoc Networks.

[42] Hamrioui S., Lorenz P. (2017). Bio inspired routing algorithm and efficient communications within IoT. IEEE Netw..

[43] Yang X.S., He X. (2013). Firefly algorithm: Recent advances and applications. arXiv.

[44] Horng M.H. (2012). Vector quantization using the firefly algorithm for image compression. Expert Syst. Appl..

[45] Chatterjee A., Mahanti G.K., Chatterjee A. (2012). Design of a fully digital controlled reconfigurable switched beam concentric ring array antenna using firefly and particle swarm optimization algorithm. Prog. Electromagn. Res..

[46] Kumbharana S.N., Pandey G.M. (2013). Solving travelling salesman problem using firefly algorithm. Int. J. Res. Sci. Adv. Technol..

[47] Anbuchelian S., Lokesh S., Baskaran M. Improving security in Wireless Sensor Network using trust and metaheuristic algorithms. Proceedings of the 2016 IEEE 3rd International Conference on Computer and Information Sciences (ICCOINS).

[48] Pirbhulal S., Shang P., Wu W., Sangaiah A.K., Samuel O.W., Li G. (2018). Fuzzy vault-based biometric security method for tele-health monitoring systems. Comput. Electr. Eng..

[49] Pirbhulal S., Zhang H., Wu W., Zhang Y.T. A comparative study of fuzzy vault based security methods for wirless body sensor networks. Proceedings of the 2016 IEEE 10th International Conference on Sensing Technology (ICST).

[50] Kazemzadeh Azad S. (2011). Optimum design of structures using an improved firefly algorithm. Iran Univ. Sci. Technol..

[51] Tuba E., Tuba M., Beko M. (2018). Two stage wireless sensor node localization using firefly algorithm. Smart Trends in Systems, Security and Sustainability.

[52] Sai V.O., Shieh C.S., Nguyen T.T., Lin Y.C., Horng M.F., Le Q.D. Parallel firefly algorithm for localization algorithm in wireless sensor network. Proceedings of the 2015 IEEE Third International Conference on Robot, Vision and Signal Processing (RVSP).

[53] Yogarajan G., Revathi T. (2018). Nature inspired discrete firefly algorithm for optimal mobile data gathering in wireless sensor networks. Wirel. Netw..

[54] Sarkar A., Murugan T.S. (2019). Cluster head selection for energy efficient and delay-less routing in wireless sensor network. Wirel. Netw..

[55] Baskaran M., Sadagopan C. (2015). Synchronous firefly algorithm for cluster head selection in WSN. Sci. World J..

[56] Hamzah A., Shurman M., Al-Jarrah O., Taqieddin E. (2019). Energy-Efficient Fuzzy-Logic-Based Clustering Technique for Hierarchical Routing Protocols in Wireless Sensor Networks. Sensors.

[57] Canovas-Carrasco S., Garcia-Sanchez A.J., Garcia-Haro J. (2018). A nanoscale communication network scheme and energy model for a human hand scenario. Nano Commun. Netw..

[58] Hellman K., Colagrosso M. (2006). Investigating a wireless sensor network optimal lifetime solution for linear topologies. J. Interconnect. Netw..

[59] Dietrich I., Dressler F. (2009). On the lifetime of wireless sensor networks. ACM Trans. Sens. Netw. (TOSN).

[60] Jornet J.M., Akyildiz I.F. (2014). Femtosecond-long pulse-based modulation for terahertz band communication in nanonetworks. IEEE Trans. Commun..

[61] Jornet J.M. A joint energy harvesting and consumption model for self-powered nano-devices in nanonetworks. Proceedings of the 2012 IEEE International Conference on Communications (ICC).

